# Determinantes da Capacidade Funcional em Pacientes com Doença de Chagas

**DOI:** 10.36660/abc.20200462

**Published:** 2021-07-27

**Authors:** Whesley Tanor Silva, Henrique Silveira Costa, Pedro Henrique Scheidt Figueiredo, Márcia Maria Oliveira Lima, Vanessa Pereira Lima, Fábio Silva Martins da Costa, Matheus Ribeiro Ávila, Vanessa Amaral Mendonça, Ana Cristina Rodrigues Lacerda, Maria Carmo Pereira Nunes, Manoel Otávio Costa Rocha

**Affiliations:** 1 Universidade Federal dos Vales do Jequitinhonha e Mucuri Faculdade de Ciências Biológicas e da Saúde Escola de Fisioterapia Diamantina MG Brasil Laboratório de Reabilitação Cardiovascular, Escola de Fisioterapia, Faculdade de Ciências Biológicas e da Saúde, Universidade Federal dos Vales do Jequitinhonha e Mucuri (UFVJM), Diamantina, MG - Brasil.; 2 Universidade Federal de Minas Gerais Hospital das Clínicas Faculdade de Medicina Belo Horizonte MG Brasil Programa de Pós-graduação em Infectologia e Medicina Tropical, Departamento de Medicina Interna, Faculdade de Medicina e Hospital das Clínicas da Universidade Federal de Minas Gerais (UFMG), Belo Horizonte, MG - Brasil.

**Keywords:** Doença de Chagas, Cardiomiopatia Chagásica, Teste de Esforço, Insuficiência Cardíaca/complicações, Tromboembolia, Trypanosoma Cruzi

## Abstract

**Fundamento:**

A doença de Chagas leva à redução da capacidade funcional. Entretanto, o estágio em que o comprometimento funcional é detectável permanece obscuro.

**Objetivos:**

O presente estudo teve como objetivo comparar a capacidade funcional de pacientes em diferentes estágios da doença de Chagas e de indivíduos saudáveis e verificar os determinantes do consumo de oxigênio de pico (VO2pico).

**Métodos:**

Em um estudo transversal, foram selecionados 160 indivíduos, 35 saudáveis e 125 com doença de Chagas. No grupo chagásico, 61 (49%) estavam na forma indeterminada da doença, 45 (36%) com cardiomiopatia chagásica (CC) e função cardíaca preservada e 19 (15%) com disfunção cardíaca e CC dilatada. Os dados foram analisados por meio de análise de regressão univariada e multivariada. A significância estatística foi fixada em 5%.

**Resultados:**

Pacientes na forma indeterminada da doença apresentaram capacidade funcional semelhante a indivíduos saudáveis (p> 0,05). Pacientes com ChC e função cardíaca preservada apresentaram VO2pico menor que os pacientes na forma indeterminada (p <0,05), mas apresentaram valores de VO2pico semelhantes ao ChC dilatado (p = 0,46). A idade, sexo masculino, classe funcional da NYHA, pressão arterial diastólica, razão entre a velocidade do fluxo transmitral diastólico precoce e a velocidade anular mitral diastólica precoce, a fração de ejeção do ventrículo esquerdo (FEVE) e o diâmetro diastólico final do ventrículo esquerdo foram associados à capacidade funcional. Porém, apenas idade, sexo masculino, FEVE e classe funcional da NYHA permaneceram associados ao VO2pico no modelo final (R2 ajustado = 0,60).

**Conclusão:**

Pacientes com CC apresentam menor capacidade funcional do que pacientes na forma indeterminada. FEVE, idade, sexo masculino e classe funcional da NYHA foram determinantes do VO2pico em pacientes com doença de Chagas.

## Introdução

A doença de Chagas, uma infecção causada pelo protozoário *Trypanosoma cruzi* , continua sendo um problema sério de saúde pública mais de 100 anos após ter sido descoberta. ^1^ A doença afeta cerca de 6 a 7 milhões de pessoas na América Latina, ^2^ com um aumento drástico em áreas não endêmicas, tais como os Estados Unidos e a Europa. ^3^^,^^4^

Na fase crônica, a maioria das pessoas mantêm-se assintomáticas, embora infectadas, na forma indeterminada da doença. Na forma indeterminada, os indivíduos infectados têm prognóstico semelhante ao de sujeitos saudáveis. ^5^ Portanto, os pacientes geralmente são chamados de pacientes assintomáticos ou chagásicos sem cardiopatia aparente. ^6^ Achados clínicos na forma indeterminada da doença incluem pequenas alterações eletrocardiográficas tais como incompetência cronotrópica, arritmias ventriculares induzidas por esforço, e alterações segmentares no ecocardiograma, sem alterações na função sistólica do ventrículo esquerdo e sem alterações eletrocardiográficas significativas. ^7^ Cerca de 30 a 40% desses pacientes desenvolvem a forma cardíaca. ^5^

A forma cardíaca, também chamada de cardiomiopatia chagásica (CCH) é a manifestação clínica mais grave e mais comum, com alterações eletrocardiográficas importantes, piora progressiva da função sistólica com dilatação ventricular. A CCH dilatada, o estágio final da doença cardíaca, pode evoluir com insuficiência cardíaca, tromboembolismo e arritmias malignas. ^8^^,^^9^

Fadiga e dispneia são achados clínicos comuns de envolvimento cardíaco ^5^ e, consequentemente, é esperada a redução da capacidade funcional e da tolerância ao exercício. Entretanto, não está claro em que estágio o comprometimento funcional pode ser detectado. Alguns autores relataram que a capacidade funcional reduzida somente pode ser detectada em CCH dilatada devido a insuficiência cardíaca. Outros demonstraram que o comprometimento funcional pode ocorrer nos estágios iniciais da cardiopatia, ^10^ até mesmo antecedendo a disfunção sistólica.

A identificação do estágio é desejável para a estratificação do risco e para a adoção de medidas preventivas eficientes. Portanto, o presente estudo foi conduzido para avaliar a capacidade funcional em vários estágios da doença de Chagas, para comparar a capacidade funcional, bem como as variáveis clínicas, demográficas e ecocardiográficas, em vários estágios da doença e em comparação com sujeitos saudáveis, e para verificar os fatores associados ao VO2pico em pacientes com doença de Chagas.

## Métodos

Este estudo transversal foi realizado no Ambulatório de Referência em Doença de Chagas e em um Laboratório de Reabilitação Cardiovascular no Brasil, entre junho de 2013 e junho de 2018. Todos os pacientes deram o consentimento informado por escrito voluntariamente antes de participar do estudo. A pesquisa foi realizada de acordo com a Declaração de Helsinki ^11^ e foi aprovada pelo comitê de ética institucional.

### Desenho do estudo

A amostra incluiu sujeitos saudáveis e pacientes com um amplo espectro de doença de Chagas. Os cálculos de tamanho da amostra post hoc foram realizados utilizando-se o software GPower, versão 3.1. Considerando que 125 sujeitos com doença de Chagas foram avaliados para conveniência, um erro alfa de 5% e 4 preditores, obteve-se um poder estatístico de 95%. Os critérios de inclusão no grupo de doença de Chagas foram a presença de dois ou mais testes sorológicos com resultado positivo para *Trypanosoma cruzi* . A amostra saudável foi composta de sujeitos sem alterações clínicas significativas ou doenças sistêmicas.

O grupo chagásico foi estratificado de acordo com a apresentação clínica (forma indeterminada, CCH com função cardíaca preservada ou CCH dilatada). Pacientes com a forma indeterminada devem apresentar uma ausência de sintomas clínicos significativos que sugerissem o comprometimento funcional devido à doença de Chagas, e um raio X do tórax com uma silhueta cardíaca normal e ECG convencional dentro dos limites de normalidade. ^12^

Os critérios de inclusão no grupo de CCH foram achados clínicos, eletrocardiográficos ou ecocardiográficos compatíveis com CCH ^9^ e condição clínica estável. Pacientes foram incluídos no grupo de CCH dilatada, quando demonstraram fração de ejeção ventricular esquerda (FEVE) abaixo de 52% (para homens) ou 54% (para mulheres) ^13^ e diâmetro diastólico final do ventrículo esquerdo (DDFVE) acima de 55mm. Os critérios de exclusão de todos os pacientes foram a presença de doença cardíaca ou sistêmica por qualquer outra causa, comorbidades associadas, e a incapacidade de realizar o teste ergométrico.

A população geral do estudo foi submetida a avaliação clínica, ecocardiografia e teste ergométrico máximo. O ecocardiograma foi realizado de acordo com as recomendações da *American Society of Echocardiography* (Sociedade Americana de Ecocardiografia). ^13^ A FEVE foi obtida pela regra de Simpson modificada. A razão entre a velocidade da onda E do fluxo mitral e da velocidade diastólica e’ do anel mitral (Relação E/e’) foi calculada. Todos os sujeitos realizaram um teste ergométrico limitado por sintomas em uma esteira (Digistress Pulsar, Micromed, Brasília, Brasil) utilizando o protocolo padrão de Bruce. O pico de consumo de oxigênio (VO2pico), que foi estimado por uma fórmula específica [VO2pico (mL/kg/min) = 2,33 (tempo em minutos) + 9,48], ^14^ foi considerada para a avaliação funcional.

### Análise estatística

A distribuição normal dos dados foi analisada utilizando-se o teste Kolmogorov-Smirnov. As variáveis contínuas foram apresentadas como média e desvio padrão (distribuição normal) ou mediana e faixa interquartil (distribuição não normal), e as variáveis categóricas foram apresentadas como número absoluto e porcentagem.

Variáveis categóricas foram comparadas pelo teste Qui-quadrado. Foram verificadas diferenças entre grupos pelo ANOVA de uma via com correções de Bonferroni, ou teste de Kruskal-Wallis com teste de comparações múltiplas de Dunn para análises post hoc, conforme apropriado. Os determinantes do VO2pico foram verificados por regressão linear univariada e multivariada reversa. As variáveis associadas ao VO2pico na análise univariada (p<0,1) foram incluídas no modelo multivariado. Na análise de regressão linear, quatro premissas foram adotadas: linearidade, distribuição de resíduos, homocedasticidade, e a ausência de multicolinearidade. A linearidade das variáveis independentes e resíduos foi verificada por gráficos de dispersão, e a distribuição de resíduos foi analisada pelo histograma. A homocedasticidade foi verificada pelo gráfico de dispersão e caracterizada pela distribuição equânime de resíduos na linha de regressão. A ausência de multicolinearidade foi definida como valores de fator de inflação de variância (VIF) abaixo de 10,0. Além disso, a autocorrelação das variáveis foi verificada pelo teste de Durbin-Watson, e valores entre 1,5 e 2,5 demonstram que não existe autocorrelação nos dados. A significância estatística foi definida em 5%. Os dados foram analisados com o software SPSS, versão 17.0 (SPSS Inc., Chicago, IL).

## Resultados

Um total de 160 indivíduos foram selecionados e avaliados: 35 (22%) indivíduos saudáveis, 61 (38%) pacientes com doença de Chagas na forma indeterminada, 45 (28%) com CCH e função cardíaca preservada, e 19 (12%) com CCH dilatada. Características demográficas, clínicas, ecocardiográficas e funcionais da amostra são apresentadas na Tabela 1 , estratificadas por apresentação clínica.

**Tabela 1:** Características demográficas, clínicas, ecocardiográficas e funcionais da amostra avaliada, estratificadas por apresentação clínica (n=160)

Variáveis		Indivíduos saudáveis (n=35)	Pacientes com doença de Chagas			p-valor *
			Forma indeterminada (n=61)	CCH e função cardíaca preservada (n=45)	CCH dilatada (n=19)	
Idade (anos)		47,0 (36.7-52.0)	43,5 (38.0-51.0)	52,0 (43.7-61.5)^a,b^	52,5 (45.7-58.2)^a,b^	**<0,001**
Sexo masculino (%)		21 (60)	28 (46)	16 (35)^a^	12 (63)^c^	0,083
IMC (kg/m²)		25,9 (23,8-29,4)	25,9 (23,6-29,3)	26,8 (23,5-29,4)	25,6 (22,2-30,9)	0,875
Classe funcional NYHA						**0,035**
	I	35 (100)	61 (100)	32 (71)	5 (26)	
	II	0	0	13 (29)	6 (32)	
	III	0	0	0	8 (42)	
PAS (mmHg)		127,3±14,7	120,0±12,7	118,7±19,8^a^	102,3±17,0^a,b,c^	**<0,001**
PAD (mmHg)		86,3±8,5	84,1±7,5	74,2±9,8^a,b^	66,5±7,0^a,b,c^	**<0,001**
FC (bpm)		69,4±7,7	72,2±11,3	71,6±18,9	64,8±11,3	0,255
Relação E/e’		5,1 (4,3 – 6,4)	5,7 (4,4 – 7,2)	8,5 (6,7 – 11,4)^a,b^	9,7 (6,8 – 12,3)^a,b^	**<0,001**
FEVE (%)		70,0±5,4	68,1±5,1	64,9±7,1^a^	38,8±7,9^a,b,c^	**<0,001**
DDFVE (mm)		47,2±5,5	48,6±4,2	48,7±4,9	62,9±10,6^a,b,c^	**<0,001**

Dados apresentados como média e desvio padrão (distribuição normal), mediana e faixa interquartil (distribuição não normal), ou número absoluto e porcentagem (variáveis categóricas). IMC: índice de massa corporal; NYHA: classe funcional da New York Heart Association; PAS: pressão arterial sistólica; PAD: pressão arterial diastólica; FC: frequência cardíaca; FEVE: fração de ejeção ventricular esquerda; DDFVE: diâmetro diastólico final do ventrículo esquerdo; Relação E/e’: razão entre a velocidade da onda E do fluxo mitral e da velocidade diastólica e’ do anel mitral.

* p-valor da comparação entre os quatro grupos por ANOVA de uma via. a, b, c representam um p-valor <0,05 verificado por análises post hoc de Bonferroni em comparação a: asujeitos saudáveis; bpacientes com doença de Chagas na forma indeterminada; ccardiomiopatia chagásica com função cardíaca preservada.

### Diferenças entre sujeitos saudáveis e pacientes com formas clínicas diferentes da doença de Chagas

Na comparação entre indivíduos saudáveis e pacientes com doença de Chagas na forma indeterminada, não houve diferença significativa em nenhuma das variáveis. Em contraste, pacientes com CCH e função cardíaca preservada eram predominantemente do sexo feminino (p=0,025), mais velhos, com classe funcional NYHA pior, valores mais baixos de pressão arterial diastólica e sistólica, capacidade funcional mais baixa, relação E/e’ mais alta, e FEVE mais baixa (p<0,001 para todos), em comparação a indivíduos saudáveis.

Pacientes com CCH dilatada eram mais velhos, com classe funcional NYHA pior, valores mais baixos de pressão arterial diastólica e sistólica, capacidade funcional mais baixa, relação E/e’ mais alta, FEVE mais baixa, e DDFVE mais alto (p<0,001 para todos), em comparação a indivíduos saudáveis.

### Diferenças demográficas, clínicas, ecocardiográficas e funcionais entre as formas clínicas da doença de Chagas

Pacientes com doença de Chagas na forma indeterminada eram mais jovens, apresentaram classe funcional NYHA melhor, pressão arterial diastólica mais alta, e relação E/e’ mais alta (p<0.001 para todos) quando comparado a CCH e função cardíaca preservada. Além disso, pacientes na forma indeterminada eram mais jovens, apresentaram classe funcional NYHA melhor, pressão arterial diastólica mais alta, relação E/e’ mais alta, FEVE mais alta e DDFVE mais baixo (p<0.001 para todos) quando comparado a pacientes com CCH dilatada.

Por último, pacientes com CCH e função cardíaca preservada são predominantemente do sexo feminino (p=0,040) quando comparado a CCH dilatada, bem como com classe funcional da NYHA, valores mais altos de pressão arterial diastólica e sistólica, FEVE mais alta e DDFVE mais baixo (p<0.001 para todos).

### Diferenças entre sujeitos saudáveis e pacientes com doença de Chagas, e entre as formas clínicas da doença de Chagas

**Figura 1:**
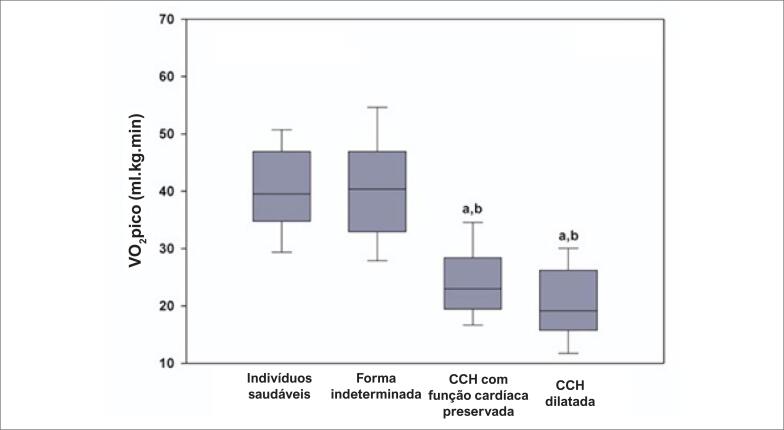
Pico de consumo de oxigênio (VO2pico) em indivíduos saudáveis e em pacientes com doença de Chagas com formas clínicas diferentes. ^a^: p<0,001 comparado a indivíduos saudáveis; ^b^: p<0.001 comparado a indivíduos com a forma indeterminada de doença de Chagas.

Os resultados da avaliação de capacidade funcional são apresentados na Figura 1 . Na população geral do estudo, houve diferenças significativas entre os grupos (p<0,001). Pacientes com a forma indeterminada da doença de Chagas tinham VO2pico semelhante aos dos sujeitos saudáveis. Pacientes com CCH e função cardíaca preservada apresentaram uma redução significativa da capacidade funcional em relação aos participantes saudáveis e os pacientes com doença de Chagas na forma indeterminada (p<0,001 para ambos), com médias de diferenças de 15,7 mL.kg.min (IC 95% 10,5 – 20,8) e 16,1 mL.kg.min (IC 95% 11,6 – 20,6), respectivamente. Por último, pacientes com CCH dilatada tiveram VO2pico mais baixo em comparação a sujeitos saudáveis e pacientes na forma indeterminada (p<0,001 para ambos), com médias de diferenças de 20,0 mL.kg.min (IC 95% 13,3 – 26,6) e 20,3 mL.kg.min (IC 95% 14,2 – 26,5), respectivamente. Não houve diferenças de VO2pico entre pacientes com CCH dilatada e com CCH e função cardíaca preservada (p=0,467).

### Determinantes do VO2pico em pacientes com doença de Chagas

Na análise univariada, idade, sexo masculino, classe funcional da NYHA, pressão arterial diastólica, relação E/e’, FEVE e DDFVE foram associados ao VO2pico. Entretanto, no modelo multivariado final, apenas idade, sexo masculino, classe funcional da NYHA e FEVE se mantiveram como determinantes do VO2pico, com R² padronizado de 0,60 ( Tabela 2 ).

**Tabela 2:** Fatores associados ao VO2pico nas análises univariada e multivariada da população Chagásica (n=125)

Variáveis	Análise univariada				Análise multivariada *			
	Coeficiente B	IC 95%	r	p-valor	Coeficiente B	IC 95%	p-valor	Estatísticas de colinearidade (valores VIF)
Constante	-	-	-	-	29,5	13,5 a 46,4	**<0,001**	
Idade (anos)	-0,6	-0,8 a -0,5	0,5	**<0,001**	-0,2	-0,5 a -0,2	**0,038**	**1,13**
Sexo masculino	10,9	7,5 a 14,3	0,5	**<0,001**	9,6	6,3 a 13,4	**<0,001**	**1,18**
IMC (kg/m^2^)	-0,3	-0,7 a -0,1	0,1	0,209	-	-	**-**	
Classe NYHA	-11,8	-14,9 a -8,6	0,5	**<0,001**	-4,2	-8,3 a -0,1	**0,041**	**1,92**
PAS (mmHg)	0,1	-0,1 a 0,1	0,1	0,352	-	-	-	
PAD (mmHg)	0,4	0,2 a 0,5	0,3	**<0,001**	-	-	-	
FC (bpm)	-0,1	-0,1 a 0,1	0,1	0,982	-	-	**-**	
Relação E/e’	-0,5	-1,5 a 0,7	0,3	**<0,001**	-	-	-	
FEVE (%)	0,5	0,3 a 0,6	0,5	**<0,001**	0,3	0,2 a 0,5	**<0,001**	**1,99**
DDFVE (mm)	-0,4	-0,6 a -0,1	0,2	**0,003**				

r: coeficiente de correlação; VE: ventrículo esquerdo; IMC: índice de massa corporal; NYHA: classe funcional da New York Heart Association; PAS: pressão arterial sistólica; PAD: pressão arterial diastólica; FC: frequência cardíaca; bpm: batimentos por minuto; Relação E/e’: relação entre velocidade diastólica precoce de fluxo de transmissão e velocidade diastólica precoce do anel mitral; FEVE: fração de ejeção ventricular esquerda; DDFVE: diâmetro diastólico final do ventrículo esquerdo; VIF: fator de inflação de variância.

* O valor R² para o modelo multivariado final foi de 0,60.

Na análise visual das premissas de regressão linear, a linearidade das variáveis independentes, a distribuição normal e a homocedasticidade dos resíduos foram verificados. O teste de Durbin-Watson demonstrou a ausência da autocorrelação nos dados (d = 1,6). Além disso, os valores de VIF destacam a ausência de multicolinearidade ( Tabela 2 ).

## Discussão

Pacientes com doença de Chagas geralmente evoluem com fadiga progressiva e dispneia, e a intolerância ao exercício é um achado clínico comum nessa população. ^15^ Entretanto, o estágio da doença em que o comprometimento funcional é detectável ainda não está claro. Os principais achados do presente estudo foram: (1) Pacientes com doença de Chagas na forma indeterminada tinham capacidade funcional semelhante à dos sujeitos saudáveis; (2) o VO2pico em pacientes com CCH era significativamente mais baixo do que em pacientes com a forma indeterminada, e (3) o FEVE, juntamente com idade, sexo masculino e classe funcional da NYHA, explicam 60% das variações da capacidade funcional. O presente estudo sugere que, mesmo sem danos miocárdicos significativos, pacientes com CCH e função cardíaca preservada têm comprometimento funcional. Esses achados são úteis para o entendimento do impacto da doença na capacidade funcional e estratificação de risco do paciente, e demonstram a importância da avaliação funcional periódica nessa população, como também ajudam na identificação de pacientes que precisam de treinamento de esforço supervisionado.

Pacientes com a forma indeterminada da doença de Chagas são reconhecidamente assintomáticos e têm um bom prognóstico de médio prazo. Entretanto, estudos demonstram que exames mais precisos, tais como testes de esforço, são capazes de detectar alterações nessa população, em comparação a sujeitos saudáveis. ^16^ Costa et al. ^17^ relataram a prevalência mais alta de arritmias ventriculares induzidas por esforço e disfunção vagal por arritmias sinusais respiratórias em pacientes indeterminados em comparação a sujeitos saudáveis. Entretanto, os autores não identificaram diferenças na capacidade funcional (p>0,05). Durante os testes ergométricos, Rocha et al. ^18^ demonstraram um aumento na prevalência de arritmias ventriculares induzidas por exercício e incompetência cronotrópica em pacientes com doença de Chagas sem doença cardíaca em comparação a sujeitos saudáveis, sem diferença na capacidade funcional (p>0,05). Da mesma forma, o presente estudo não identificou diferenças na capacidade funcional entre os dois grupos. Acredita-se que alterações subclínicas possam estar presentes em pacientes com a forma indeterminada da doença de Chagas, porém, sem alterações na capacidade ergométrica.

Por outro lado, pacientes com a forma cardíaca da doença, tanto com função cardíaca preservada quanto com disfunção ventricular, apresentaram redução da função sistólica, função diastólica, e capacidade funcional em relação a pacientes com a forma indeterminada da doença de Chagas e a indivíduos saudáveis. Vários estudos não conseguiram determinar o estágio da doença em que o comprometimento funcional é detectável. Um estudo anterior demonstrou que a redução da capacidade funcional ocorre nos estágios iniciais da doença cardíaca. ^10^ Outros estudos demonstraram que o comprometimento funcional é detectável em pacientes com doença de Chagas apenas na presença de cardiomiopatia avançada. ^19^ Recentemente, uma revisão sistemática com meta-análise ^15^ relatou que o comprometimento funcional ocorre na CCH, mesmo em pacientes com função ventricular preservada. Entretanto, essa análise incluiu poucos estudos e os resultados devem ser interpretados com cautela. Poucos estudos incluíram as principais formas de doença de Chagas em um único manuscrito. Nossos resultados são consistentes com os da análise sistemática, demonstrando que pacientes com CCH e função cardíaca preservada tinham valores de VO2pico e FEVE mais baixos do que os apresentados por indivíduos saudáveis e pacientes com doença de Chagas na forma indeterminada, até mesmo com valores dentro de limites normais. A CCH dilatada apresentou VO2pico mais baixo que indivíduos saudáveis e todas as outras formas de doença de Chagas.

Além disso, os resultados deste estudo apresentaram uma redução na função diastólica em pacientes com CCH e função cardíaca preservada, em comparação com os grupos de forma indeterminada e com indivíduos saudáveis, o que poderia levar a uma redução do VO2pico. Na verdade, a relação E/e’ foi associada ao VO2pico em análise univariada. Entretanto, ela não foi mantida no modelo multivariado final. Portanto, parece que a função diastólica, embora reduzida no grupo com CCH e função cardíaca preservada, não é um determinante de capacidade funcional em pacientes com doença de Chagas.

O presente estudo também demonstrou os fatores associados à capacidade funcional em pacientes com doença de Chagas. A FEVE é um determinante de capacidade funcional, e, juntamente com idade, sexo masculino e classe funcional da NYHA, explica 60% das variações de VO2pico. Idade e sexo são preditores bem estabelecidos de capacidade funcional na população geral. Há uma relação inversamente proporcional entre idade e capacidade ergométrica, assim como a mulheres tendem a ter um VO2pico mais baixo do que os homens. ^20^^–^^22^ Na verdade, a massa muscular e a força podem ser reduzidas em 30% a 50% entre os 30 e os 80 anos, pela perda de fibra muscular e atrofia da fibra muscular tipo II. ^23^^,^^24^ Em relação ao sexo, as mulheres têm câmaras ventriculares esquerdas menores e volumes sistólicos mais baixos, ^25^ enchimento diastólico mais baixo, ^26^ maior prevalência de obesidade ^25^ e menor massa magra do que os homens, ^27^ o que poderia explicar a menor capacidade ergométrica.

Vários estudos não conseguiram demonstrar uma relação entre FEVE e capacidade funcional, ^28^^,^^29^ relatando que outros fatores, tais como função ventricular direita e átrio esquerdo, são mais relacionados a exercício que à FEVE. Entretanto, outro estudo encontrou diferenças significativas em pacientes com CCH e FEVE preservada e disfunção ventricular, ^30^ já que tanto o VO2pico quanto a FEVE tendem a diminuir com o avanço da doença. Acredita-se que a redução da FEVE leva à má perfusão da musculatura esquelética durante o exercício, ^31^ causando fadiga e dispneia, e contribuindo para a intolerância ao exercício. Entretanto, são necessários estudos mais detalhados para confirmar a hipótese.

O presente estudo tem limitações e pontos fortes. Uma limitação do estudo foi a realização do teste de esforço utilizando-se os testes ergométricos máximos, sem gasometria. Estabeleceu-se que a avaliação indireta do VO2pico está correlacionado à medição direta, ^32^ enquanto outros autores relataram uma discrepância considerável entre os valores de VO2pico estimados e avaliados. ^33^ Apesar dos resultados conflitantes, deve-se destacar que áreas endêmicas da doença de Chagas geralmente têm poucos recursos tecnológicos e, de acordo com uma revisão sistemática recente, 77% dos estudos com objetivo de verificar a capacidade funcional na população utilizaram a medição indireta do VO2pico sem gasometria. Portanto, acredita-se que o uso do VO2pico estimado para a avaliação funcional é uma limitação, mas não invalida os resultados, especialmente considerando-se o contexto da doença de Chagas. Além disso, a amostra foi composta por pacientes monitorados em um ambulatório de referência no tratamento de doenças parasitárias, que são avaliados regularmente e estão em tratamento otimizado. Apesar da importância dos achados dessa população negligenciada, os resultados podem não refletir a capacidade funcional de todos os pacientes com doença de Chagas, especialmente os que estão em área endêmica. Além disso, a análise intra e interobservador na avaliação da capacidade funcional não foi verificada. Entretanto, todos os testes foram realizados por apenas dois cardiologistas experientes, certificados pela Sociedade Brasileira de Cardiologia, o que possivelmente reduziu o viés e pode não ter alterado os resultados da avaliação funcional. Por último, o presente estudo incluiu apenas um parâmetro de função diastólica (relação E/e’), e é necessário verificar se outras variáveis de função diastólica estão associadas à capacidade funcional dessa população. Como ponto forte, o presente estudo foi o primeiro a demonstrar a FEVE como um determinante de capacidade funcional. Além disso, a redução significativa de VO2pico em pacientes com CCH, comparada com a de pacientes com a forma indeterminada da doença, sugere que pacientes com CCH, independentemente da função cardíaca, devem passar por treinamento ergométrico supervisionado para evitar o comprometimento funcional grave.

## Conclusão

Pacientes com CCH, mesmo com função ventricular preservada, tinham menor capacidade funcional que pacientes com a forma indeterminada. Em pacientes com doença de Chagas, FEVE, idade, sexo masculino e classe funcional da NYHA são determinantes da capacidade funcional.
