# Supplementary material for: Determinantes da Capacidade Funcional em Pacientes com Doença de Chagas
Source: Arq Bras Cardiol. 2021 Jul 27;117(5):934–41. [Article in Portuguese] doi: 10.36660/abc.20200462 (PMC8682109; doi:10.36660/abc.20200462)
Supplement: Translation

# Translation: Determinants of Functional Capacity in Patients with Chagas Disease

**Authors:** Whesley Tanor Silva, Henrique Silveira Costa, Pedro Henrique Scheidt Figueiredo, Márcia Maria Oliveira Lima, Vanessa Pereira Lima, Fábio Silva Martins da Costa, Matheus Ribeiro Ávila, Vanessa Amaral Mendonça, Ana Cristina Rodrigues Lacerda, Maria Carmo Pereira Nunes, Manoel Otávio Costa Rocha

**Affiliations:** 1 Diamantina MG Brazil Laboratório de Reabilitação Cardiovascular, Escola de Fisioterapia, Faculdade de Ciências Biológicas e da Saúde, Universidade Federal dos Vales do Jequitinhonha e Mucuri (UFVJM), Diamantina, MG - Brazil.; 2 Belo Horizonte MG Brazil Programa de Pós-graduação em Infectologia e Medicina Tropical, Departamento de Medicina Interna, Faculdade de Medicina e Hospital das Clínicas da Universidade Federal de Minas Gerais (UFMG), Belo Horizonte, MG - Brazil.

**Keywords:** Chagas Disease, Chagas Cardiomyopathy, Exercise Test/methods/métodos, Heart Failure/complications, Thromboembolism, Trypanosoma Cruzi

## Abstract

**Background:**

Chagas disease leads to reduced functional capacity. However, the stage at which functional impairment is detectable remains unclear.

**Objectives:**

The present study was addressed to compare the functional capacity of patients at different stages of Chagas disease and healthy individuals and to verify the determinants of peak oxygen uptake (VO2peak).

**Methods:**

In a cross-sectional study, 160 individuals were selected, 35 healthy and 125 with Chagas disease. In the Chagasic group, 61 (49%) were in the indeterminate form of the disease, 45 (36%) with Chagas cardiomyopathy (ChC) and preserved cardiac function and 19 (15%) with cardiac dysfunction and dilated ChC. The data were analyzed using univariate and multivariate regression analysis. Statistical significance was set at 5%.

**Results:**

Patients in the indeterminate form of disease showed similar functional capacity to healthy individuals (p>0.05). Patients with ChC and preserved cardiac function had lower VO2peak than patients in the indeterminate form (p<0.05), but showed similar VO2peak values than dilated ChC (p=0.46). The age, male sex, NYHA functional class, diastolic blood pressure, ratio of the early diastolic transmitral flow velocity to early diastolic mitral annular velocity, left ventricular ejection fraction (LVEF) and left ventricular end-diastolic diameter were associated with functional capacity. However, only age, male sex, LVEF and NYHA functional class, remained associated with VO2peak in the final model (adjusted R2=0.60).

**Conclusion:**

Patients with ChC had lower functional capacity than patients in the indeterminate form. LVEF, age, male sex and NYHA functional class were determinants with VO2peak in patients with Chagas disease.

## Introduction

Chagas disease, an infection caused by the protozoan *Trypanosoma cruzi* , still remains a serious public health problem more than 100 years after its discovery. ^1^ The disease affects about six to seven million people in Latin America, ^2^ with a dramatic increase in non-endemic areas such as the United States and Europe. ^3^^,^^4^

Most people remain asymptomatic in the chronic phase but infected in the indeterminate form of the disease. In the indeterminate form, infected individuals have similar prognosis to healthy subjects. ^5^ Therefore, patients are usually referred to as asymptomatic or Chagasic patients without apparent cardiopathy. ^6^ Clinical findings in the indeterminate form include minor echo or electrocardiographic changes, such as chronotropic incompetence, exercise-induced ventricular arrhythmias, and segmental changes on the echocardiogram, with no changes in left ventricular systolic function and with no significant electrocardiographic changes. ^7^ About 30% to 40% of these patients will develop the cardiac form. ^5^

The cardiac form, also called Chagas cardiomyopathy (ChC), is the most common and severe clinical manifestation, with important electrocardiographic changes, progressive worsening of the systolic function with ventricular dilation. Dilated ChC, the end stage of heart disease, may evolve with heart failure, thromboembolism, and malignant arrhythmias. ^8^^,^^9^

Fatigue and dyspnea are common clinical findings of cardiac involvement ^5^ and, consequently, the reduction in functional capacity and exercise tolerance is expected. However, it remains unclear at what stage functional impairment can be detected. Some authors have reported that the reduced functional capacity is detectable only in dilated ChC due to heart failure. Others demonstrated that functional impairment may occur in the early stages of cardiopathy, ^10^ even preceding systolic dysfunction.

The stage identification is desirable for risk stratification and the adoption of effective preventive measures. Thus, the present study was addressed to evaluate the functional capacity in different stages of Chagas disease in order to compare the functional capacity, as well as clinical, demographic, and echocardiographic variables, at different stages of the disease, as compared to healthy patients, and to verify the factors associated with VO2peak in patients with Chagas disease.

## Methods

This cross-sectional study was conducted at the Referral Outpatient Center for Chagas Disease and at a Cardiovascular Rehabilitation Laboratory, Brazil, between June 2013 and June 2018. All the patients voluntarily gave their written informed consent prior to participating in this study. The research was carried out in accordance with the Declaration of Helsinki ^11^ and was approved by the institutional ethics committee.

### Study design

The sample was comprised of healthy subjects and patients with a wide spectrum of Chagas disease. The post hoc sample size calculations were performed using the software GPower, version 3.1. Considering that 125 subjects with Chagas disease were evaluated for convenience, an alpha error of 5% and 4 predictors, a statistical power of 95% was obtained. The criteria for inclusion in the Chagas disease group were the presence of two or more positive serological tests for *Trypanosoma cruzi* . The healthy sample consisted of subjects without significant clinical changes or systemic diseases.

The Chagasic group was stratified according to the clinical presentation (indeterminate form, ChC with preserved cardiac function or dilated ChC). Patients in the indeterminate form should present an absence of significant clinical symptoms suggestive of functional impairment due to Chagas disease and a chest X-ray with a normal cardiac silhouette and conventional ECG within the normal limits. ^12^

Criteria for inclusion for ChC were clinical, electrocardiographic, or echocardiographic findings compatible with ChC ^9^ and a stable clinical condition. Patients were included in the dilated ChC group when they demonstrated a left ventricular ejection fraction (LVEF) of lower than 52% (for men) or 54% (for women) ^13^ and a left ventricular end-diastolic diameter (LVDd) of higher than 55mm. Exclusion criteria for all patients were the presence of systemic or heart disease by any other causes, associated comorbidities, and the inability to perform exercise testing.

The overall study population underwent clinical evaluation, echocardiography, and a maximal exercise test. Echocardiography was performed according to recommendations of the American Society of Echocardiography. ^13^ LVEF was obtained through the modified Simpson´s rule. Early diastolic velocity (e’) at the medial border of the mitral annulus was obtained and the ratio between peak mitral E and e’ (E/e’ ratio) was calculated. All subjects performed a symptom-limited exercise test on a treadmill (Digistress Pulsar, Micromed, Brasilia, Brazil), using the standard Bruce protocol. Peak oxygen uptake (VO2peak), which was estimated by a specific formula [VO2peak (mL/kg/min) = 2.33 (time in min) + 9.48], ^14^ was considered for functional evaluation.

### Statistical analysis

The normal distribution of data was assessed by the Kolmogorov-Smirnov test. Continuous variables were shown as mean and standard deviation (normal distribution) or median and interquartile range (non-normal distribution), while categorical variables were demonstrated as absolute number and percentage.

Categorical variables were compared by the Chi-squared test. Differences among groups were verified by one-way ANOVA with Bonferroni corrections or the Kruskall Wallis test with Dunn’s multiple comparison test for post-hoc analyses, as appropriate. The determinants of VO2peak were verified by univariate and backward multivariate linear regression. The variables associated with VO2peak in the univariate analysis (p<0.1) were included in the multivariate model. In the linear regression analysis, four assumptions were adopted: linearity, distribution of residuals, homoscedasticity, and the absence of multicollinearity. The linearity of the independent variables and residuals was checked by scatter plots and the distribution of residuals was analyzed by the histogram. The homoscedasticity was verified by the scatter plot and characterized by the residuals equally distributed in the regression line. The absence of multicollinearity was defined as the variance inflation factor (VIF) values below 10.0. Additionally, the auto-correlation of the variables was verified by the Durbin-Watson test and values between 1.5 and 2.5 show that there is no auto-correlation in the data. Statistical significance was set at 5%. Data were analyzed with SPSS version 17.0 (SPSS Inc., Chicago, IL, USA).

## Results

A total of 160 individuals were selected and evaluated: 35 (22%) healthy individuals, 61 (38%) patients with Chagas disease in the indeterminate form, 45 (28%) with ChC and preserved cardiac function, and 19 (12%) with dilated ChC. Demographic, clinical, echocardiographic, and functional features of the sample are presented in Table 1 , stratified by clinical presentation.

**Table 1:** Demographic, clinical, echocardiographic, and functional characteristics of the evaluated sample stratified by clinical presentation (n=160)

Variables		Healthy individuals (n=35)	Patients with Chagas disease			p-value *
			Indeterminate form (n=61)	ChC and preserved cardiac function (n=45)	Dilated ChC (n=19)	
Age (years)		47.0 (36.7-52.0)	43.5 (38.0-51.0)	52.0 (43.7-61.5)^a,b^	52.5 (45.7-58.2)^a,b^	**<0.001**
Male sex (%)		21 (60)	28 (46)	16 (35)^a^	12 (63)^c^	0.083
BMI (kg/m^2^)		25.9 (23.8-29.4)	25.9 (23.6-29.3)	26.8 (23.5-29.4)	25.6 (22.2-30.9)	0.875
NYHA functional class						**0.035**
	I	35 (100)	61 (100)	32 (71)	5 (26)	
	II	0	0	13 (29)	6 (32)	
	III	0	0	0	8 (42)	
SBP (mmHg)		127.3±14.7	120.0±12.7	118.7±19.8^a^	102.3±17.0^a,b,c^	<0.001
DBP (mmHg)		86.3±8.5	84.1±7.5	74.2±9.8^a,b^	66.5±7.0^a,b,c^	**<0.001**
HR (bpm)		69.4±7.7	72.2±11.3	71.6±18.9	64.8±11.3	0.255
E/e’ ratio		5.1 (4.3 – 6.4)	5.7 (4.4 – 7.2)	8.5 (6.7 – 11.4)^a,b^	9.7 (6.8 – 12.3)^a,b^	**<0.001**
LVEF (%)		70.0±5.4	68.1±5.1	64.9±7.1^a^	38.8±7.9^a,b,c^	**<0.001**
LVDd (mm)		47.2±5.5	48.6±4.2	48.7±4.9	62.9±10.6^a,b,c^	**<0.001**

Data presented as mean and standard deviation (normal distribution). median and interquartile range (non-normal distribution) or number and percentage (categorical variables). BMI: body mass index; NYHA: New York Heart Association functional class; SBP: systolic blood pressure; DBP: diastolic blood pressure; HR: heart rate; LVEF: left ventricular ejection fraction; LVDd: left ventricular end-diastolic diameter; E/e’ ratio: ratio of the early diastolic transmitral flow velocity to early diastolic mitral annular velocity.

* p-value of the comparison among the four groups by One-way ANOVA. a. b. c represent a p-value < 0.05 verified by Bonferroni post-hoc analyses when compared to: ahealthy subjects; bpatients with Chagas disease in the indeterminate form; cChagas cardiomyopathy with preserved cardiac function.

### Differences between healthy subjects and patients in different clinical forms of Chagas disease

In the comparison between healthy individuals and patients with Chagas disease in the indeterminate form, there was no significant difference in any variables. In contrast, patients with ChC and preserved cardiac function were predominantly female (p=0.025), older, with worse NYHA functional class, lower values of systolic and diastolic blood pressure, lower functional capacity, higher E/e’ ratio, and a lower LVEF (p<0.001 for all), when compared to healthy individuals.

Patients with dilated ChC were older, with a worse NYHA functional class, lower values of systolic and diastolic blood pressure, lower functional capacity, higher E/e’ ratio, lower LVEF, and higher LVDd (p<0.001 for all), when compared to healthy individuals.

### Demographic, clinical, echocardiographic, and functional differences among the clinical forms of Chagas disease

Patients with Chagas disease in the indeterminate form were younger, showed better NYHA functional class, higher diastolic blood pressure, and a lower E/e’ ratio (p<0.001 for all), when compared to ChC and preserved cardiac function. Additionally, patients in the indeterminate form were younger, showed a better NYHA functional class, higher systolic and diastolic blood pressures, a lower E/e’ ratio, a higher LVEF and a lower LVDd (p<0.001 for all), when compared to patients with dilated ChC.

Finally, patients with ChC and preserved cardiac function are predominantly female (p=0.040), when compared to dilated ChC, as well as with a better NYHA functional class, higher values of systolic and diastolic blood pressure, a higher LVEF, and a lower LVDd (p<0.001 for all).

### Functional differences between healthy subjects and patients with Chagas disease, and among the clinical forms of Chagas disease

The results of the functional capacity assessment are shown in Figure 1 . In the overall study population, significant differences were found among the groups (p<0.001). Patients in the indeterminate form of Chagas disease presented a VO2peak that was similar to healthy patients. Patients with ChC and preserved cardiac function showed a significant reduction in functional capacity in relation to healthy participants and patients with Chagas disease in the indeterminate form (p<0.001 for both), with a mean of difference of 15.7 mL.kg.min (95% CI 10.5 – 20.8) and 16.1 mL.kg.min (95% CI 11.6 – 20.6), respectively. Finally, patients with dilated ChC had a lower VO2peak when compared to healthy subjects and patients in the indeterminate form (p<0.001 for both), and the mean differences were of 20.0 mL.kg.min (95% CI 13.3 – 26.6) and 20.3 mL.kg.min (95% CI 14.2 – 26.5), respectively. No difference was found in VO2peak between patients with dilated ChC and with ChC and preserved cardiac function (p=0.467).

**Figure 1:** Peak oxygen uptake (VO2peak) in healthy individuals and in patients with Chagas disease with different clinical forms.^a^: p<0.001 compared to healthy individuals;^b^: p<0.001 compared to the indeterminate form of Chagas disease.

### Determinants of VO2peak in patients with Chagas disease

In the univariate analysis, age, male sex, NYHA functional class, diastolic blood pressure, E/e’ ratio, LVEF, and LVDd were associated with the VO2peak. However, in the final multivariable model, only age, male sex, NYHA functional class, and LVEF remained as determinants of the VO2peak, with an adjusted R ^2^ of 0.60 ( Table 2 ).

**Table 2:** Factors associated with the VO2peak in the uni and multivariate analysis of the Chagasic population (n=125)

Variables	Univariate analysis				Multivariate analysis *			
	B-coefficient	95% CI	r	p-valor	B-coefficient	95% CI	p-value	Collinearity statistics (VIF values)
Constant	-	-	-	-	29.5	13.5 to 46.4	**<0.001**	
Age (years)	-0.6	-0.8 a -0.5	0.5	**<0.001**	-0.2	-0.5 to -0.2	**0.038**	**1.13**
Male sex	10.9	7.5 a 14.3	0.5	**<0.001**	9.6	6.3 to 13.4	**<0.001**	**1.18**
BMI (kg/m^2^)	-0.3	-0.7 to -0.1	0.1	0.209	-	-	**-**	
NYHA class	-11.8	-14.9 a -8.6	0.5	**<0.001**	-4.2	-8.3 to -0.1	**0.041**	**1.92**
SBP (mmHg)	0.1	-0.1 to 0.1	0.1	0.352	-	-	-	
DBP (mmHg)	0.4	0.2 a 0.5	0.3	**<0.001**	-	-	-	
HR (bpm)	-0.1	-0.1 a 0.1	0.1	0.982	-	-	**-**	
E/e’ ratio	-0.5	-1.5 a 0.7	0.3	**<0.001**	-	-	-	
LVEF (%)	0.5	0.3 a 0.6	0.5	**<0.001**	0.3	0.2 a 0.5	**<0.001**	**1.99**
LVDd (mm)	-0.4	-0.6 to -0.1	0.2	**0.003**				

r: correlation coefficient; LV: left ventricular; BMI: body mass index; NYHA: New York Heart Association functional class; SBP: systolic blood pressure; DBP: diastolic blood pressure; HR: heart rate; bpm - beats per minute; E/e’ ratio: ratio of the early diastolic transmitral flow velocity to early diastolic mitral annular velocity; LVEF: left ventricular ejection fraction; LVDd: left ventricular end-diastolic diameter; VIF: variance inflation factor.

* The R2 value for the final multivariate model was 0.60.

In the visual analysis of the linear regression assumptions, the linearity of the independent variables, the normal distribution and the homoscedasticity of the residuals were verified. The Durbin-Watson test demonstrated the absence of auto-correlation in the data (d = 1.6). In addition, VIF values highlight the absence of multicollinearity ( Table 2 ).

## Discussion

Patients with Chagas disease usually evolve with progressive fatigue and dyspnea, and exercise intolerance is a common clinical finding in this population. ^15^ However, the stage of the disease at which functional impairment is detectable remains unclear. The main findings of the present study were: (1) Chagas disease patients in the indeterminate form had similar functional capacity to healthy patients; (2) the VO2peak in patients with ChC was significantly lower than in patients in the indeterminate form; and (3) the LVEF, together with age, male sex, and NYHA functional class, explains 60% of the variations in functional capacity. The present study suggests that, even without significant myocardial damage, patients with ChC and preserved cardiac function have functional impairment. These findings are useful in understanding the impact of the disease in the functional capacity and risk stratification of the patient, and demonstrate the importance of periodic functional assessment in this population, as well as assist in identifying patients who need supervised exercise training.

Patients in the indeterminate form of Chagas disease are known to be asymptomatic and have a good medium-term prognosis. However, studies have shown that more accurate examinations, such as exercise testing, are able to detect changes in this population when compared to healthy individuals. ^16^ Costa et al. ^17^ reported the higher prevalence of exercise-induced ventricular arrhythmias and vagal dysfunction by respiratory sinus arrhythmias in indeterminate patients versus healthy subjects. However, the authors found no difference in functional capacity (p>0.05). During exercise testing, Rocha et al. ^18^ demonstrated an increased prevalence of exercise-induced ventricular arrhythmias and chronotropic incompetence in patients with Chagas disease without heart disease, as compared to healthy subjects, with no difference in the functional capacity (p>0.05). Similarly, the present study also found no difference in functional capacity between the two groups. It is believed that subclinical changes may be present in patients in the indeterminate form of Chagas disease but without changes in exercise capacity.

On the other hand, patients in the cardiac form of the disease, both with preserved cardiac function and with ventricular dysfunction, showed a reduction in the systolic function, diastolic function, and functional capacity in relation to patients in the indeterminate form of Chagas disease and healthy individuals. Many studies have failed to determine the stage of the disease at which functional impairment is detectable. A previous study has shown that the reduction of functional capacity occurs in the early stages of heart disease. ^10^ Another study demonstrated that functional impairment is detectable in patients with Chagas disease only in the presence of advanced cardiomyopathy. ^19^ Recently, a systematic review with meta-analysis ^15^ reported that functional impairment occurs in ChC, even in patients with preserved ventricular function. However, this review included few studies and the results should be interpreted with caution. Few studies have included the main forms of Chagas disease in a single manuscript. Moreover, our results are consistent with the systematic review, showing that patients with ChC and preserved cardiac function presented lower a VO2peak and LVEF values than healthy individuals and patients with Chagas disease in the indeterminate form, even with values within normal limits. Dilated ChC showed lower VO2peak than healthy individuals and all other forms of Chagas disease.

In addition, our results showed a reduction in the diastolic function in patients with ChC and preserved cardiac function when compared to the indeterminate and healthy groups, which could lead to a reduction in VO2peak. In fact, the E/e’ ratio was associated with the VO2peak in the univariate analysis; however, it did not remain in the final multivariate model. Thus, it seems that the diastolic function, although reduced in the group with ChC and preserved cardiac function, is not a determinant of functional capacity in patients with Chagas disease.

The present study also demonstrated the factors associated with functional capacity in patients with Chagas disease. The LVEF is a determinant of functional capacity, and together with age, male sex, and NYHA functional class, it explains 60% of variations in the VO2peak. Age and sex are well-established predictors of functional capacity in the general population. There is an inverse relationship between age and exercise capacity, just as women tend to have a lower VO2peak than men. ^20^^–^^22^ In fact, muscle mass and strength can be reduced by 30% to 50% between 30 and 80 years of age by the loss of muscle fibers and atrophy of the type II muscle fiber. ^23^^,^^24^ Regarding sex, women have smaller left ventricular chambers and lower stroke volumes, ^25^ lower diastolic compliance, ^26^ greater prevalence of obesity, ^25^ and less lean mass than men, ^27^ which would explain the lower exercise capacity.

Regarding LVEF, many studies failed to demonstrate an association between LVEF and functional capacity, ^28^^,^^29^ reporting that other factors, such as right ventricular function and left atrium, are more related to exercise than LVEF. However, another study has found significant differences in patients with ChC and preserved LVEF and ventricular dysfunction, ^30^ since both the VO2peak and LVEF tend to decrease with disease progression. It is believed that the reduction in LVEF leads to poor skeletal muscle perfusion during exercise, ^31^ causing fatigue and dyspnea, and contributing to exercise intolerance. However, further studies are needed to confirm the hypothesis.

The present study has limitations and strengths. One limitation of this study was the performance of the stress test using conventional maximal exercise testing, without gas analysis. The indirect assessment of the VO2peak has been established to be correlated with the direct measurement, ^32^ while other authors have reported a considerable discrepancy between the estimated and evaluated VO2peak values. ^33^ Despite the conflicting results, it is emphasized that the endemic areas in Chagas disease generally have few technological resources and, according to a recent systematic review, 77% of the studies that aimed to verify the functional capacity in this population used the indirect measure of the VO2peak without gas analysis. Therefore, we believe that the use of the estimated VO2peak for functional assessment is a limitation, but it does not invalidate the results, especially considering the setting of Chagas disease. In addition, our sample consisted of patients followed up by a referral center for the treatment of parasitic diseases, and they are regularly evaluated and are undergoing optimized therapy. Despite the importance of the findings of this neglected population, the results may not reflect the functional capacity of all patients with Chagas disease, especially those in an endemic area. Moreover, the intra and interobserver analysis in the assessment of functional capacity was not verified. However, all tests were performed by only two experienced cardiologists, certified by the Brazilian Society of Cardiology, which possibly reduced the bias and may not have changed the results of the functional assessment. Finally, the present study included only one parameter of diastolic function (E/e’ ratio), which is necessary to verify whether other diastolic function variables are associated with the functional capacity of this population. As a strength, the present study was the first that demonstrated the LVEF as a determinant of functional capacity. Furthermore, the significant reduction in the VO2peak in patients with ChC,a s compared to those in the indeterminate form, suggests that patients with ChC, regardless of cardiac function, should undergo supervised exercise training to prevent severe functional impairment.

## Conclusion

Patients with ChC, even with preserved ventricular function, presented a lower functional capacity than did patients in the indeterminate form. In patients with Chagas disease, LVEF, age, male sex, and NYHA functional class are determinants of functional capacity.
